# Comparison of direct machine parameter optimization versus fluence optimization with sequential sequencing in IMRT of hypopharyngeal carcinoma

**DOI:** 10.1186/1748-717X-2-33

**Published:** 2007-09-06

**Authors:** Barbara Dobler, Fabian Pohl, Ludwig Bogner, Oliver Koelbl

**Affiliations:** 1 Department of Radiotherapy, University of Regensburg, Regensburg, Germany

## Abstract

**Background:**

To evaluate the effects of direct machine parameter optimization in the treatment planning of intensity-modulated radiation therapy (IMRT) for hypopharyngeal cancer as compared to subsequent leaf sequencing in Oncentra Masterplan v1.5.

**Methods:**

For 10 hypopharyngeal cancer patients IMRT plans were generated in Oncentra Masterplan v1.5 (Nucletron BV, Veenendal, the Netherlands) for a Siemens Primus linear accelerator.

For optimization the dose volume objectives (DVO) for the planning target volume (PTV) were set to 53 Gy minimum dose and 59 Gy maximum dose, in order to reach a dose of 56 Gy to the average of the PTV. For the parotids a median dose of 22 Gy was allowed and for the spinal cord a maximum dose of 35 Gy. The maximum DVO to the external contour of the patient was set to 59 Gy. The treatment plans were optimized with the direct machine parameter optimization ("Direct Step & Shoot", DSS, Raysearch Laboratories, Sweden) newly implemented in Masterplan v1.5 and the fluence modulation technique ("Intensity Modulation", IM) which was available in previous versions of Masterplan already. The two techniques were compared with regard to compliance to the DVO, plan quality, and number of monitor units (MU) required per fraction dose.

**Results:**

The plans optimized with the DSS technique met the DVO for the PTV significantly better than the plans optimized with IM (p = 0.007 for the min DVO and p < 0.0005 for the max DVO). No significant difference could be observed for compliance to the DVO for the organs at risk (OAR) (p > 0.05). Plan quality, target coverage and dose homogeneity inside the PTV were superior for the plans optimized with DSS for similar dose to the spinal cord and lower dose to the normal tissue. The mean dose to the parotids was lower for the plans optimized with IM. Treatment plan efficiency was higher for the DSS plans with (901 ± 160) MU compared to (1151 ± 157) MU for IM (p-value < 0.05).

Renormalization of the IM plans to the mean of the dose to 95% of the PTV (D_95_) of the DSS plans, resulted in similar target coverage and dose to the parotids for both strategies, at the cost of a significantly higher dose to the normal tissue and maximum dose to the target. The relative volume of the PTV receiving 107% or more of the prescription dose V_107 _increased to 35.5% ± 20.0% for the IM plan as compared to a mean of 0.9% ± 0.9% for the DSS plan.

**Conclusion:**

The direct machine parameter optimization is a major improvement compared to the fluence modulation with subsequent leaf sequencing in Oncentra Masterplan v1.5. The resulting dose distribution complies better with the DVO and better plan quality is achieved for identical specification of DVO. An additional asset is the reduced number of MU as compared to IM.

## Background

In the treatment planning of radiation therapy of hypopharyngeal cancer the major challenge is to spare the spinal cord and preserve the function of the parotid glands without compromising the dose to the target [1-8]. Because the parotid glands are often in close proximity to the target and the spinal cord is located in a concavity of the target this can be best achieved by intensity modulated radiation therapy (IMRT) [9-14].

Various treatment planning systems with different optimization algorithms are commercially available for IMRT. Some of them use the optimization of fluence matrices, which have to be converted in deliverable MLC segments after optimization. Because of limitations of the MLC settings the resulting fluence is different from the optimization result and therefore no longer optimal [15]. Other systems incorporate the MLC sequencing in the optimization process [16,17], or optimize the machine parameters directly [18,19]. In both cases the MLC position is taken into account in the optimization process and the resulting optimal fluence can be delivered by the linac without further approximations [15]. This is usually refered to as direct machine parameter optimization (DMPO) or direct aperture optimization (DAO) [20-29].

The aim of this study is to compare the direct machine parameter optimization versus fluence optimization with subsequent leaf sequencing for IMRT of hypopharyngeal carcinoma with respect to compliance with the DVO, efficiency and plan quality.

## Methods

### Patients

10 patients with hypopharyngeal cancer, 9 male and 1 female, were included in the planning study.

### Equipment

Treatment planning was performed with the treatment planning system (TPS) Oncentra Masterplan^® ^v1.5 SP1 (Nucletron BV, Veenendal, the Netherlands) on a Siemens Primus linear accelerator (linac) with a photon energy of 6 MV and a double focused multileaf collimator (MLC) with 29 leaf pairs with 1 cm resolution at isocenter for the 27 inner leaf pairs and 6.5 cm for the two outer leaf pairs. Since the two outer leaf pairs are not taken into account by the optimization module and the maximum overtravel of the leaves is 10 cm, the maximally useable field size for IMRT is 20 cm × 27 cm.

The TPS Oncentra Masterplan v1.5 has two options for the optimization process, both products of RaySearch Laboratories AB, Sweden: In the so called "Intensity Modulation" (IM) option the optimization is performed for the energy fluence of the beams and the MLC segments are created afterwards in a separate leaf sequencing process. The user can define a maximal number of segments and the sequencer will iteratively create a number of segments as close as possible and below or equal to the predefined maximum. The final dose calculation is performed based on these segments. In the "Direct Step and Shoot" (DSS) option a fluence optimization with subsequent leaf sequencing as described above is performed for a few iterations to get an initial guess for the segments. In the next step, the gradients of the objective function are calculated with respect to leaf positions and weights, which allows to optimize the MLC segments directly. The result of this optimization are MLC segments ready for delivery without further post-processing. This is also known as direct machine parameter optimization [15]. A detailed description can be found in [19].

Other parameters regarding the MLC segments which can be chosen by the user include the minimum number of monitor units per segment and fraction, the minimum number of adjacent open leaf pairs and the minimum size of a segment.

### Structure definition

The planning target volume (PTV) in the first series up to a prescribed dose of 56 Gy encompassed in all patients the primary tumor site in the hypopharynx and the adjuvant lymphatics [30,31] (supraclavicular, jugulodigastric, upper and middle jugular chain, midcervical, submaxillary, spinal accessory and retropharyngeal lymph nodes (RPLN) = Level II-VI + RPLN). Because of the propensity of hypopharynx cancer to spread submucosally the PTV expands from base of scull to the upper cervical esophagus as described in "Principles and Practice of Radiation Oncology" [32]. Facing the free communication with both sides of lymphatic drainage in all cases both sides of the neck were enclosed in the PTV. As organs at risk the parotid gland on both sides and the spinal cord were delineated.

### Treatment goals

IMRT optimization was performed on the PTV with a goal dose of 56 Gy to the average of the PTV. Since there is no option to define a DVO for the average dose in the TPS, the minimum and maximum DVO for the PTV were set symmetrically to the desired average dose, i.e. the minimum DVO to 53 Gy representing 95% of the goal and the maximum DVO to 59 Gy representing 105% of the goal dose. For the parotid glands no more than 50% of the volume were allowed to receive more than 22 Gy or 39% of the goal dose, and the maximal dose for the spinal cord was chosen to be 35 Gy or 63% of the goal dose. The DVO to the organs at risk were chosen relatively low with respect to the additional dose given by the boost treatment or other dose prescription schemes. For a total prescription dose of 70 Gy this would correspond to a max dose of 44 Gy to the spinal cord and 27.5 Gy to no more than 50% of the parotids which complies with the RTOG protocol 0022 for IMRT for oropharyngeal cancer. An overview over the DVO used in this study is given in table 1.

**Table 1:** Dose Volume Objectives (DVO) and weights used for optimization.

**Structure**	**DVO type**	**weight**	**Dose in Gy**	**Dose in %**	**Volume in %**
**PTV**	min	3000	53	95	100
	max	3000	59	105	0
**left parotid**	max	300	22	39	50
**right parotid**	max	300	22	39	50
**spinal cord**	max	300	35	63	0
**External**	max	3000	60	107	0

### Radiation technique

A 7 field coplanar treatment plan with beam angles of 0°, 51°, 103°, 154°, 206°, 257°, 308° was generated in Masterplan v1.5 with a photon energy of 6 MV for each patient. Using the dose volume objectives given in table 1, the plan was optimized with the DSS option first. The maximal number of segments was set to 70 – 100 depending on the patient geometry and the complexity of the structures. The parameter "minimal open field size" which limits the minimal size of each segment was set to 4 cm^2^, the minimal number of adjacent open leaf pairs to 2, and the minimal number of MU per fraction and segment to 4. Dose calculation was performed using the pencil beam algorithm with inhomogeneity correction and a dose grid resolution of 0.4 cm^3^. The maximum number of iterations in the optimization process was set to 50 – 70.

The weight for the DVO of the PTV and the external structure was primarily set to 3000, for organs at risk to 300. During the optimization process the weights and number of segments were adapted slightly in some cases in order to improve the result.

Once a satisfying result was achieved, a second plan was created and optimized with the IM option using identical optimization parameters. The dose was normalized to the average dose of the PTV, the prescription dose was the goal dose of 56 Gy.

### Evaluation

Since one objective of the study was to quantify the quality of the optimization algorithm, treatment plans were evaluated after optimization (and segmentation for IM) and final dose calculation without performing any additional renormalization to the goal dose. As a measure for how good the DVO were fulfilled by the respective optimization strategy, absolute dose differences between the DVO and the corresponding dose volume histogram (DVH) points of the treatment plans were calculated and compared for the two optimization strategies. The differences are given in absolute values for DVH points which violate the DVO. For DVH points which fulfill the DVO, the difference value is set to 0. For the PTV D_95 _and D_5 _were used for comparison to the minimum and maximum DVO to account for the fact that part of the PTV is located in the build-up region and to avoid evaluation of cold and hot spots of very small volumes. To assess the efficiency of the treatment, the number of monitor units (MU) and segments were reported and compared.

For evaluation of the plan quality the dose volume histograms were analyzed with regard to target coverage, dose homogeneity and OAR sparing. For the PTV the isodoses encompassing 95% and 5% of the volume D_95 _and D_5_, the average dose D_average_, and the volumes V_95 _and V_107 _covered by 95% and 107% of the prescription dose D_prescibed _of 56 Gy were computed. Target dose homogeneity was quantified using the gradient of the DVH of the PTV H = (D_5 _- D_95_)/D_average_, target coverage using V_95 _[33]. For the OAR the median dose D_50 _to the parotid glands and the maximum dose D_max _to the spinal cord were recorded.

To investigate if simple renormalisation of the two competing treatment plans could improve the plan with the poorer quality such that it would become comparable to the better plan, the evaluation of the DVH was also performed for renormalization of the treatment plans.

For statistical analysis a paired samples t-test was performed in SPSS 13.0, the significance level was chosen to be 0.05.

Dosimetric validation of the plans was beyond the scope of this planning study and was therefore not included in the manuscript.

## Results

Mean values and standard deviations of the dose differences between DVO and corresponding DVH points are given in table 2. Significant differences between the IM and the DSS optimized plans can be observed for the PTV and external contour (p-value < 0.05). The minimum DVO for the PTV was violated by the IM optimization with a mean dose difference of 3.4 Gy ± 2.7 Gy, the maximum DVO by 1.1 Gy ± 0.4 Gy, and the maximum DVO for the external contour by 4.3 Gy ± 1.3 Gy. The violations of these DVO by the DSS optimization were lower, i.e. 0.5 Gy ± 0.5 for the min DVO of the PTV, 0.1 Gy ± 0.1 Gy for the max DVO of the PTV, and 2.2 Gy ± 1.3 Gy for the maximum DVO of the external contour.

**Table 2:** Comparison of plan compliance to the DVO

	**IM**		**DSS**		
	mean	SD	mean	SD	p-value

**PTV**					
Dmin/D95	3.4	2.7	0.5	0.5	0.007
Dmax/D5	1.1	0.4	0.1	0.1	< 0.0005
**left parotid**					
D50	0.2	0.7	0.6	1.0	0.4
**right parotid**					
D50	0.2	0.4	0.3	0.5	0.6
**spinal cord**					
Dmax	0.0	0.1	0.0	0.0	0.3
**external**					
Dmax	4.3	1.3	2.2	1.3	0.001

Mean values and standard deviations of the dose differences (in Gy) between DVO and corresponding DVH points for the plans optimized with IM and DSS for all patients. Positive values are used for DVH points which violate the DVO. For DVH points which fulfill the DVO, the difference values are set to 0. Significant differences between the IM and the DSS optimized plans can be observed for the PTV and external contour (p-value < 0.05). For the parotids and the spinal cord no significant differences can be observed for the two optimization strategies.

For the parotids and the spinal cord no significant differences were observed for the two optimization strategies (p-value > 0.05). The mean values of DVO violations were for the left parotid 0.2 Gy ± 0.7 Gy (IM) and 0.6 Gy ± 1.0 Gy (DSS), for the right parotid 0.2 Gy ± 0.4 Gy (IM) and 0.3 Gy ± 0.5 Gy (DSS), and for the spinal cord 0.0 Gy ± 0.1 Gy (IM) and 0.0 Gy ± 0.0 Gy (DSS).

Treatment plan efficiency was higher for the DSS plans with (901 ± 160) MU per 2 Gy fraction compared to (1151 ± 157) MU for IM (p-value < 0.05). The number of segments was in the same range for both optimization strategies (77 ± 8).

**Figure 1:**
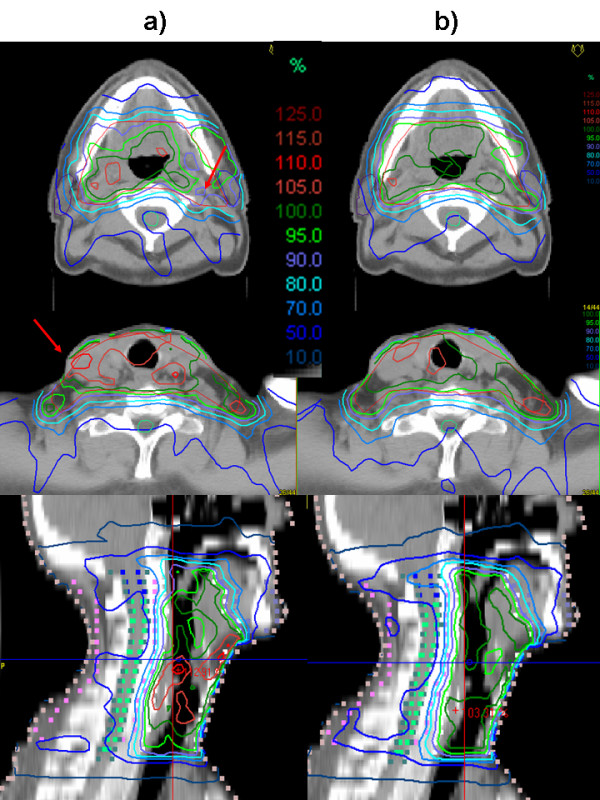
Isodoses of the plans optimized with a) IM and b) DSS for one of the patients in two representative transversal slices and the central sagittal plane. The better target coverage is visible particularly in the region around the right parotis. The red arrows point out regions of underdosage in the plan optimized with IM.

**Figure 2:**
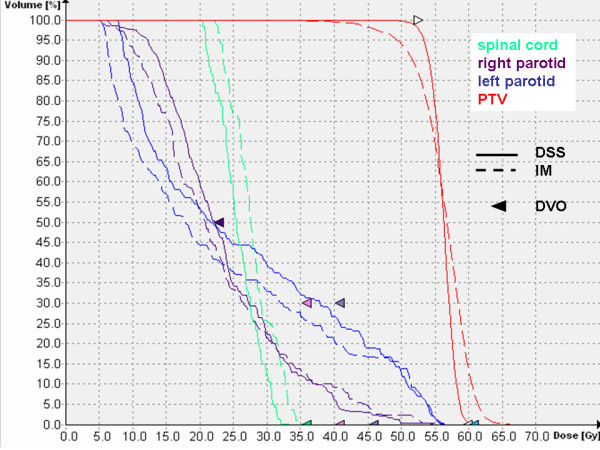
Comparison of the DVH of the plans optimized with IM and DSS for one of the patients. The DVH of the PTV show a better target coverage and homogeneity for the plan optimized with DSS. The DVH of the parotids illustrates the compliance to the DVO of both plans, indicated by the purple arrow.

Figure 1 shows a comparison of the isodoses generated with IM and DSS for two representative transversal slices and the central sagittal plane of one of the patients. Figure 2 shows the corresponding DVH. Mean values, standard deviations and p-values of selected DVH points of all patients are given in table 3. The evaluation of the treatment plan quality by means of DVH showed a significant difference for the PTV coverage and homogeneity (p < 0.05). Target coverage given by the mean value of V_95 _was significantly lower for IM plans (81.0% ± 8.3%) than for the DSS plans (91.9% ± 3.3%) (p = 0.002). V_107 _was larger for IM (6.7% ± 2.5%) than for DSS (0.9% ± 0.9%), with a p-value p < 0.0005. The mean value for the homogeneity, given by the relative dose difference H = (D_5 _- D_95_)/D_average _of the DVH of the PTV, was higher for the IM plan (18.9% ± 5.4%) than for the DSS (10.8% ± 1.7%, p < 0.0005), which means the DVH was steeper and a significantly more homogeneous dose distribution inside the target could be achieved with DSS. D_average _was in the same range for both techniques with 55.7 Gy ± 1.0 Gy (IM) and 56.0 Gy ± 0.2 Gy (DSS).

**Table 3:** Comparison of plan quality for the plans resulting from the optimization

	**IM**		**DSS**		**p-value**
		
**PTV**	mean	SD	mean	SD	
**PTV**					
D95	49.6	2.7	52.6	0.6	0.005
D5	60.1	0.4	58.6	0.4	< 0.0005
D average	55.7	1.0	56.0	0.2	0.3
H = (D_5_-D_95_)/D_average_	18.9	5.4	10.8	1.7	< 0.0005
V95*	81.0	8.3	91.9	3.3	0.002
V107*	6.7	2.5	0.9	0.9	< 0.0005
**left parotid**					
D50	19.0	2.4	22.0	1.6	0.007
**right parotid**					
D50	20.4	1.8	21.9	0.9	0.03
**spinal cord**					
Dmax	31.1	2.9	30.5	3.2	0.4
**external**					
Dmax	64.3	1.3	62.2	1.3	0.001
**Efficiency**					
# Segments	77.0	7.9	76.6	7.9	0.9
# MU	1151	157	901	160	0.007

Mean values, standard deviations and p-values for the treatment plans resulting from the optimization with IM and DSS respectively. Dose values are given in Gy, the homogeneity H in % of the average dose and volumes in % of the volume of interest.

The dose to the parotids was lower for the IM optimized plans than for the DSS plans with a mean dose of 19.0 Gy ± 2.4 Gy (IM) and 22.0 Gy ± 1.6 Gy (DSS) for the left parotid (p = 0.007) and 20.4 Gy ± 1.8 Gy (IM) and 21.9 Gy ± 0.9 Gy (DSS) for the right parotid (p = 0.03). The maximum dose to the spinal cord was comparable in both cases with 31.1 Gy ± 2.9 (IM) and 30.5 Gy ± 3.2 Gy (DSS). The maximum dose to the external contour was higher for IM (64.3 Gy ± 1.3 Gy) than for DSS (62.2 Gy ± 1.3 Gy).

Renormalization of the IM plans to a D_95 _of 52.6 Gy (the mean of the DSS plans) did improve target coverage of the IM plans to a V_95 _of 93.4% ± 1.5 with a mean dose to the parotids still below the DVO of 22 Gy (20.2 Gy ± 2.8 Gy and 21.7 Gy ± 1.6 Gy). However, V_107 _increased at the same time to 35.5% ± 20.0% and the maximum dose to the external contour to 68.4 Gy ± 5.2 Gy. The number of MU required for one fraction increased to 1233 ± 233, i.e. to the 1.4 fold of the DSS technique. Mean values, standard deviations, and p-values of the renormalised plans are listed in table 4.

**Table 4:** Comparison of plan quality for the renormalized plans

	**IM**		**DSS**		**p-value**
		
	mean	SD	mean	SD	
**PTV**					
D95	52.6	0	52.6	0.6	1.0
D5	63.9	3.9	58.6	0.4	0.002
V95*	93.4	1.5	91.9	3.3	0.2
V107*	35.5	20.0	0.9	0.9	0.001
**left parotid**					
D50	20.2	2.8	22.0	1.6	0.06
**right parotid**					
D50	21.7	1.6	21.9	0.9	0.6
**spinal cord**					
Dmax	33.1	3.1	30.5	3.2	0.02
**external**					
Dmax	68.4	5.2	62.2	1.3	0.003
**Efficiency**					
# MU	1233	233	901	160	0.003

Mean values, standard deviations and p-values for the resulting treatment plans renormalized to a D_95 _of 52.6 Gy, which is the mean of the D_95 _of the DSS plans. Dose values are given in Gy, volumes in % of the volume of interest.

## Discussion

The plans optimized with the DSS technique met the DVO for the PTV and external contour significantly better than the plans optimized with IM, with higher target coverage and dose homogeneity inside the target and lower dose to the external contour. For the organs at risk, no significant difference could be observed with regard to violations of the DVO. The plans optimized with IM resulted in even lower dose to the parotids than required by the DVO, the DVO for the parotids were more than fulfilled at the cost of PTV coverage, dose homogeneity and dose to the normal tissue, which were violated.

**Figure 3:**
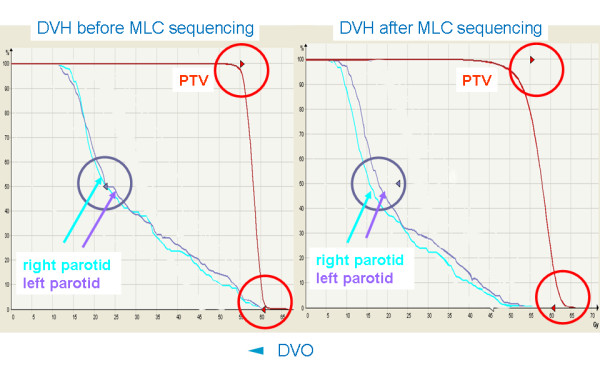
Comparision of the DVH of a plan optimized with IM before and after MLC sequencing. The DVO for the parotids (purple arrow) and PTV (red arrows) are closely met before MLC sequencing (left hand side). After MLC sequencing (right hand side) the DVH of the PTV becomes shallower, the DVO are severely violated. At the same time the median dose to the parotids, which was close to the DVO before segmentation, becomes lower after segmentation, over-fullfilling the DVO.

This can be explained by the fact, that in IM the optimization result is an optimized fluence which has to be converted into deliverable MLC segments by subsequent leaf sequencing afterwards. This sequencing process decreases the fluence levels and leads to a dose distribution which is further away from the original optimization result, the DVH smear out. In the cases studied here this leads to a lower dose to the parotids, a lower minimal dose to the PTV and a higher maximal dose to the PTV. In the DSS optimization the segments are optimized directly, the fluence resulting from the optimization process can be delivered without any further approximations and the optimal dose distribution can be achieved. Figure 3 shows a comparison of the DVH of the result of an IM optimization before and after MLC sequencing. It shows that the DVO of the PTV and the parotids are closely met before segmentation. After segmentation the DVH of the PTV becomes shallower, i.e. less homogeneous, resulting in a lower minimum dose and a higher maximum dose to the PTV. At the same time the median dose to the parotids, which was close to the DVO before segmentation, becomes lower after segmentation, over-fullfilling the DVO.

Renormalization could not improve the IM plan, since simple renormalization only shifts the DVH along the dose axis but cannot change the steepness of the DVH. Thus, target coverage can be improved, but this will at the same time always cause higher maximum dose and higher dose to the other organs.

## Conclusion

The direct machine parameter optimization is a major improvement compared to the fluence modulation with subsequent leaf sequencing in Oncentra Masterplan. The resulting dose distribution complies better with the DVO and better plan quality is achieved for identical specification of DVO. An additional asset is the reduced number of MU as compared to IM leading to a more efficient treatment delivery with less integral dose.

## Abbreviations

DSS Direct Step & Shoot

DVH Dose Volume Histogram

DVO Dose Volume Objectives

IM Intensity Modulation: Optimization with subsequent sequencing

IMRT Intensity Modulated Radiation Therapy

MLC Multi Leaf Collimator

MU Monitor Units

PTV Planning Target Volume

TPS Treatment Planning System

## Competing interests

The author(s) declare that they have no competing interests.

## Authors' contributions

BD conceived of and designed the study, carried out the planning study, evaluated the results and drafted the manuscript. FP delineated the target volumes and organs at risk and revised the manuscript, LB proof-read the manuscript, OK participated in the design of the study and revised the manuscript. All authors read and approved the final manuscript.
